# Emerging SARS-CoV-2 Diversity Revealed by Rapid Whole-Genome Sequence Typing

**DOI:** 10.1093/gbe/evab197

**Published:** 2021-08-25

**Authors:** Ahmed M Moustafa, Paul J Planet

**Affiliations:** 1 Division of Pediatric Infectious Diseases, Children’s Hospital of Philadelphia, Pennsylvania, USA; 2 Division of Gastroenterology, Hepatology, and Nutrition, Children’s Hospital of Philadelphia, Pennsylvania, USA; 3 Department of Pediatrics, Perelman College of Medicine, University of Pennsylvania, Philadelphia, Pennsylvania, USA; 4 Sackler Institute for Comparative Genomics, American Museum of Natural History, New York, New York, USA

**Keywords:** COVID-19, nomenclature, lineages, wgMLST, clonal complex, machine learning

## Abstract

Discrete classification of SARS-CoV-2 viral genotypes can identify emerging strains and detect geographic spread, viral diversity, and transmission events. We developed a tool (GNU-based Virus IDentification [GNUVID]) that integrates whole-genome multilocus sequence typing and a supervised machine learning random forest-based classifier. We used GNUVID to assign sequence type (ST) profiles to all high-quality genomes available from GISAID. STs were clustered into clonal complexes (CCs) and then used to train a machine learning classifier. We used this tool to detect potential introduction and exportation events and to estimate effective viral diversity across locations and over time in 16 US states. GNUVID is a highly scalable tool for viral genotype classification (https://github.com/ahmedmagds/GNUVID) that can quickly classify hundreds of thousands of genomes in a way that is consistent with phylogeny. Our genotyping ST/CC analysis uncovered dynamic local changes in ST/CC prevalence and diversity with multiple replacement events in different states, an average of 20.6 putative introductions and 7.5 exportations for each state over the time period analyzed. We introduce the use of effective diversity metrics (Hill numbers) that can be used to estimate the impact of interventions (e.g., travel restrictions, vaccine uptake, mask mandates) on the variation in circulating viruses. Our classification tool uncovered multiple introduction and exportation events, as well as waves of expansion and replacement of SARS-CoV-2 genotypes in different states. GNUVID classification lends itself to measures of ecological diversity, and, with systematic genomic sampling, it could be used to track circulating viral diversity and identify emerging clones and hotspots.

**Significance**

A detailed understanding of the diversity of SARS-CoV-2 is critical for identifying emerging variants and monitoring dynamic spread around the globe. We developed a method that can rapidly and systematically classify hundreds of thousands of variants of SARS-CoV-2, and we use this tool to show multiple waves of expansion and replacement of viral variants over the first months of the pandemic, including multiple introductions of virus both internationally and from more local sources. Our tool allows straightforward measurements of the diversity of the virus in a given locale over time, which may reflect the impact of real-world control strategies such as travel restrictions and mask-wearing.

## Introduction

Rapid sequencing of the SARS-CoV-2 pandemic virus has presented an unprecedented opportunity to track the evolution of the virus and to understand the emergence of a new pathogen in near-real time. During its explosive radiation and global spread, the virus has accumulated enough genomic diversity that we can identify distinct lineages and track their spread in distinct geographic locations and over time (Bedford et al. 2020; Chen et al. 2020; Deng et al. 2020; Rambaut et al. 2020; Shen et al. 2020; Worobey et al. 2020). Phylogenetic analyses in combination with rapidly growing databases (Shu and McCauley 2017; Rambaut et al. 2020) have been instrumental in identifying distinct clades and tracing how they have spread across the globe, as well as estimating calendar dates for the emergence of certain clades (Bedford et al. 2020; Deng et al. 2020; Fountain-Jones et al. 2020; Rambaut et al. 2020; Worobey et al. 2020). This information is extremely useful in assessing the impact of early measures to combat spread as well as identifying missed opportunities (Korber et al. 2020; Worobey et al. 2020).

Although reconstructing a robust phylogeny of viral variants is an intuitive approach for viral classification, traditional phylogenetic approaches suffer from problems with scalability. Building comprehensive phylogenetic trees for single nucleotide polymorphism (SNP) based analysis of SARS-CoV-2 is already extremely computationally expensive, and will become more so as hundreds of thousands of sequences are added. Additionally, although temporal effective population size of SARS-CoV-2 can be modeled using Bayesian methods such as the skygrowth package and skygrid coalescent models, they are computationally expensive for large data sets (Volz and Didelot 2018; Hill and Baele 2019; Fountain-Jones et al. 2020). Dividing the data set into subsets of genomes for analysis necessarily loses information and explanatory power.

Sequence typing tools developed so far such as Pangolin, Nextclade, Genome Detective Coronavirus Typing Tool, and COVID-19 Genotyping Tool (CGT; Hadfield et al. 2018; Cleemput et al. 2020; Maan et al. 2020; Rambaut et al. 2020; O’Toole et al. 2021) have been critical for tracking variants and communicating about the spread of certain lineages. However, the scalability of these tools is limited by computationally expensive steps (alignment and phylogenetic construction), and reliance on nonautomated curation for identifying lineages.

Because of these computational roadblocks, our goal was to develop a rapid way to categorize genomes that scales readily and leads to as little information loss as possible. We saw an opportunity to combine our allele identifying tool, WhatsGNU (Moustafa and Planet 2020b), with the multilocus sequence typing (MLST) approach (Maiden et al. 1998) that has been widely used in bacterial classification, and occasionally in viral classification (e.g., Equine Herpesvirus 1 and SARS-CoV [Wang et al. 2005; Garvey et al. 2019; Sutton et al. 2019]). MLST efficiently compresses genomic information into a form that can be easily used to reconstruct phylogenetic relationships. Our approach uses whole-genome MLST (wgMLST) to assign an allele number to each of the ten open reading frames (ORFs) in the virus’s genome creating a sequence type (ST), which is codified as the sequence of allele numbers for each of the ten genes in the viral genome. Each allele number is an exact match at the nucleotide level such that any nucleotide changes are coded as a new allele number. Thus, STs can be thought of as haplotypes that differ from each other by at least one allele. The STs are then clustered into bigger groups which are designated clonal complexes (CCs) based on their grouping on a minimum spanning tree (MST).

Here, we show that this approach allows us to link STs into clearly defined CCs that are consistent with phylogeny and other SARS-CoV-2 typing systems (Shu and McCauley 2017; Rambaut et al. 2020) using minimal computational power. Using this classification system, we measure the number of introductions and exportations of the virus in 16 US states and perform a temporal assessment of ST/CC diversity, uncovering waves of expansion and decline of distinct STs and CCs, and the apparent replacement of certain CCs with emerging lineages.

## Results and Discussion

**Fig. 1.**
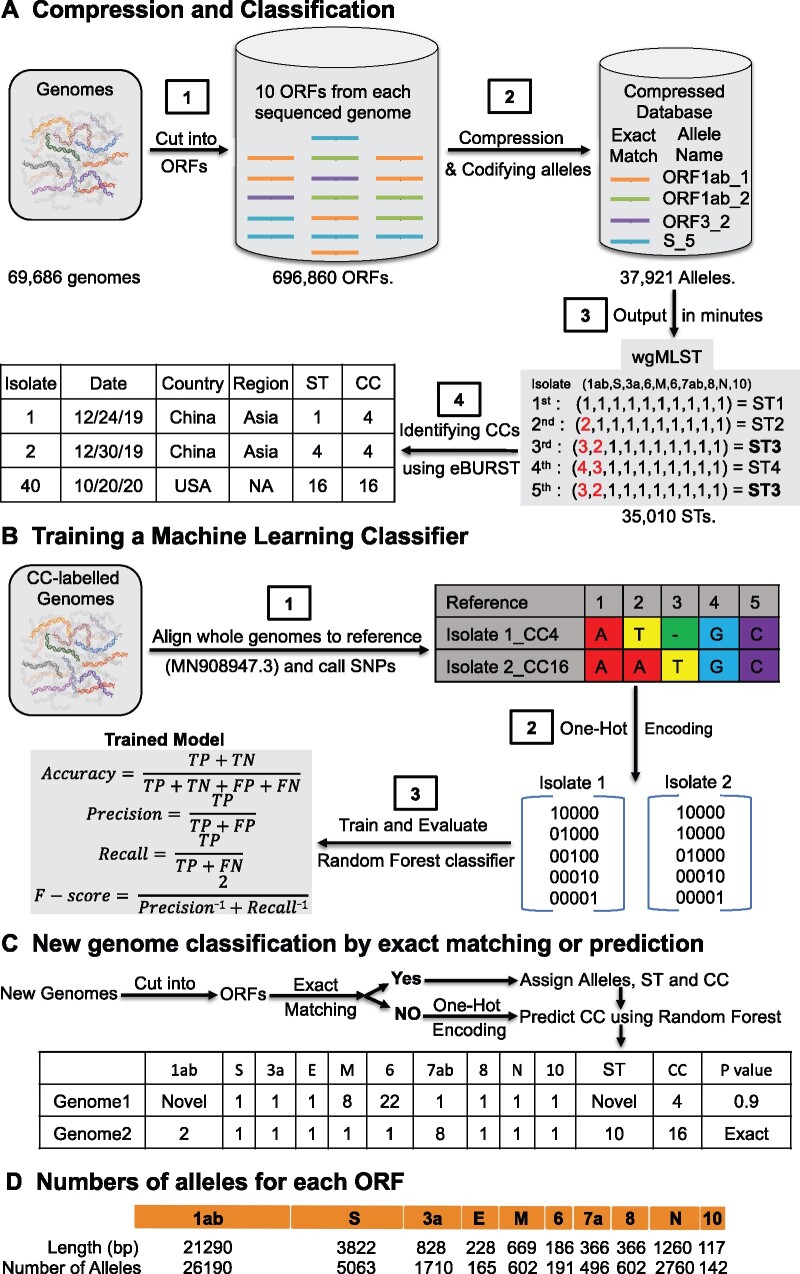
Workflow for the GNUVID tool and its compression technique. (*A*) Compression and classification. The tool starts by compressing the database of the ten ORFs of each of the SARS-CoV-2 genomes to only include a unique sequence for each allele type. The tool then uses a wgMLST approach by assigning an allele number to each gene nucleotide sequence in the virus’s genome creating an ST, which is codified as the sequence of allele numbers for each of the ten genes in the viral genome. The STs are then linked into clearly defined CCs using goeBURST. (*B*) Training a machine learning classifier. The CC-labeled genomes are then aligned to the SARS-CoV-2 reference genome (MN908947.3) and SNPs are called. The SNP matrix is then one-hot encoded and used to train a random forest classifier. The training followed a 5-fold cross-validation approach to assess the prediction capabilities of GNUVID according to four statistics (accuracy, precision, recall, and F-score). TP, TN, FP, and FN are true positives, true negatives, false positives, and false negatives, respectively. (*C*) New Genome classification by exact matching or prediction. GNUVID first tries to match each of the ten ORFs from a query SARS-CoV-2 genome to an exact match in the compressed database to define an ST, and matches that to any associated CC. If no exact match is found due to novelty or ambiguity in any of the ten ORFs, the query genome is aligned to the reference, one-hot encoded and a CC is predicted by the trained classifier. A report is then created showing the allele number of each ORF, ST, CC, and a probability of membership in the CC. (*D*) Map of SARS-CoV-2 virus genome showing the length in base pairs (bp) of the ten ORFs and numbers of alleles in the current database 69,686 isolates. The majority of the identified 37,921 unique alleles (69%) are for ORF1ab which represents 71% of the genome length. Strikingly, the two highest ratios (number of alleles/ORF length) are for the nucleocapsid protein (2.2) and ORF3a (2.1) whereas the spike protein had a ratio of 1.32. The numbers of genomes and alleles in the figure are from GNUVID v2.0.

We developed the GNU-based Virus IDentification (GNUVID) system to automatically assign a number to each unique allele of the ten ORFs of SARS-CoV-2 (Wu et al. 2020; fig. 1*A*). Beginning our analysis in October 2020, GNUVID v2.0 compressed the 696,860 ORFs in 69,686 high-quality GISAID genomes (supplementary table 1, Supplementary Material online) to 37,921 unique alleles in 5 min on a standard desktop, achieving 18-fold compression and losing no information. To create an ST for each isolate, GNUVID automatically assigned 35,010 unique ST numbers based on their allelic profile (supplementary table 1, Supplementary Material online). We then used an MST to group STs into larger taxonomic units, CCs, which we define here as clusters of >20 STs that are single or double allele variants away from a “founder.” A founder is a specific ST located at a node in the most parsimonious MST with a large number of dependent single and double locus variants (DLVs; Feil et al. 2004). Founders represent central nodes in the phylogeny and the putative ancestral genotype of the CC. Phylogenetically, a CC is ideally equivalent to a lineage of genomically related haplotypes (STs) that have diversified recently from a founder. Using the goeBURST algorithm (Feil et al. 2004; Francisco et al. 2009) to build the MST and identify founders, we found 154 CCs (fig. 1*A* and supplementary table 1, Supplementary Material online).

To benchmark the speed of GNUVID against other techniques, we assessed each step in the pipelines with a data set of all high-quality GISAID CC-labeled genomes from the GNUVID August database (25,807; table 1). GNUVID database building, MST construction, and ST/CC classification were 300 times faster than construction of a maximum likelihood (ML) phylogenetic tree from an SNP matrix employing an HKY substitution model. In addition, computing the MST with the compressed ST GNUVID data set was at least seven times faster than using an SNP matrix to build an MST. Moreover, supplementary figure 1, Supplementary Material online shows that GNUVID has scaled very well as databases have increased in size and processed almost 1 million sequences in less than 2 days on a single processor.

**Table 1:** Time (min) Needed to Process (Compress or Build Phylogenetic Tree of) 25,807 Genomes on One Processor/16 Gb RAM

Step	GNUVID	ML (8,744 SNPs)	MST (8,744 SNPs)
ORF identification by BLAST	8.8	—	—
Whole-genome alignment (minimap2)	—	4.1	4.1
Compression/wgMLST	1.5	—	—
SNP calling	—	1.8	1.8
MST Phyloviz	1.0	—	Failed due to memory
MST grapetree	3.3	—	71 (47 Gb RAM)
IQ-Tree ML Tree	—	3,570	—
Total	11	3,576	77

ORF, open reading frame; SNP, single nucleotide polymorphism.

One critical function in all classification schemes is the assignment of new sequences to groups. As mentioned before, existing tools for classifying sequences are limited by speed, scalability, and taxonomic granularity. For instance, the Genome Detective Coronavirus Typing Tool takes 1 min per genome, allows only 2,000 genomes per batch, and only assigns genomes to one of five variants of concern (B.1.1.7, B.1.351, P.1, B.1.1.70, or mink cluster; Cleemput et al. 2020). The CGT limits the number of sequence inputs to ten and can take up to 15 min per genome because of the concurrent processing of public data (Maan et al. 2020). Nextclade (Hadfield et al. 2018), a very fast tool, only classifies genomes into 12 possible clades. The Pangolin classification tool (Rambaut et al. 2020; O’Toole et al. 2021) has the most granular taxonomic scheme for classification, and a relatively fast classification approach. We compare its performance with GNUVID below.

One reason why GNUVID is so fast is its reliance on exact matching of nucleotide sequences, which is almost instantaneous. However, it is likely that many newly sequenced genomes will not find exact match of an ST even in a very large data set. Therefore, we needed a method to quickly classify new sequences without an exact match. For this goal, we used a random forest classifier trained on 53,565 CC-labeled genomes. The overall prediction statistics of the model were the weighted area under the receiver operating characteristic curve (ROC AUC; 0.987), Matthews correlation coefficient (0.953), accuracy (0.955), F-score (0.950), precision (0.947), and recall (0.964) (fig. 1*B*). Two other supervised learning algorithms, decision tree and logistic regression, were explored. Logistic regression had a lower accuracy of 0.72. The decision tree approach performed as well as random forest (accuracy: 0.957, F-score: 0.952, precision: 0.948, and recall: 0.964). However, previous work showing that probability estimates from decision trees tend to be too extreme (either 1 or 0) led us to choose a random forest (an ensemble of decision trees) approach (Margineantu and Dietterich 2003; Niculescu-Mizil and Caruana 2005; Chawla 2006).

For any new query genome, GNUVID attempts to classify it first by exact matching of the allelic profile to one of the other STs because this is most efficient and nearly instantaneous. If there is no exact match, the CC for the query genome is predicted using the trained model developed above. This query process saves time and also allows each ORF to be typed and tallied individually (fig. 1*C and D*).

The Pangolin classifier (Rambaut et al. 2020; O’Toole et al. 2021) uses a machine learning approach that greatly enhances its speed and offers a taxonomic granularity similar to the GNUVID system. Therefore, we chose to benchmark our tool against Pangolin. As expected, using 1,000 exact match genomes, GNUVID outperformed Pangolin, with a 61% reduction in processing time (31 vs 80 s), in table 2 and supplementary table 1, Supplementary Material online. The GNUVID random forest classifier was only slightly faster using 1,000 genomes without an exact match (table 2 and supplementary table 1, Supplementary Material online), but had the added benefit of assigning an allele number for each individual ORF. In downstream analysis, allele numbers could be used to quickly find amino acid residues of concern or define emerging variants.

**Table 2:** Prediction Time (s) for 1,000 Genomes by GNUVID and Pangolin

Genomes	GNUVID	GNUVID	Pangolin
	(Exact Matching and Random Forest)	(Random Forest)	(Decision Tree)
Exact match	31	52	80
New	69	51	81

To show that CCs are mostly consistent with whole-genome phylogenetic trees, we mapped the ten most common CC designations onto an ML tree. Members of the same CC visually grouped together in clades (supplementary fig. 2, Supplementary Material online). To further validate our classification system, we compared it to the proposed “dynamic lineages nomenclature” for SARS-CoV-2 used in Pangolin (Rambaut et al. 2020) and the GISAID clade naming system (Shu and McCauley 2017). A high percentage of CCs, 95.5% (147/154) and 87.7% (135/154) of the CCs, had 90% of their genomes assigned to the same GISAID clade and Pangolin lineage, respectively, showing strong agreement between these classification schemes (supplementary table 1, Supplementary Material online).

One limitation of our classification strategy, as with many schemes that operate in real time, is that paraphyletic groups can occur as a new ST arises from an older ST (e.g., CC258 and CC768 emerged from CC255 and CC258 making CC255 and CC258 paraphyletic, respectively) (supplementary fig. 2, Supplementary Material online). Paraphyly and misclassification can be quantified as the consistency of a classification scheme with a phylogenetic tree. Thus, to better quantify the phylogenetic consistency of our classification system and compare it to an existing tool like Pangolin, we mapped the GNUVID CCs and Pangolin lineages as character states for 25,170 genomes on a tree constructed using an SNP matrix, independently (Lanfear and Mansfield. 2020). We then calculated the retention index (Farris 1989) for each classification scheme. The retention index calculates the amount of homoplasy on a tree on a scale of 0 to 1, where 1 is a perfect match with the phylogeny and 0 is complete disagreement with the tree. Both GNUVID and Pangolin showed high retention indices (0.915 and 0.97, respectively) reflecting low levels of disagreement.

**Fig. 2.**
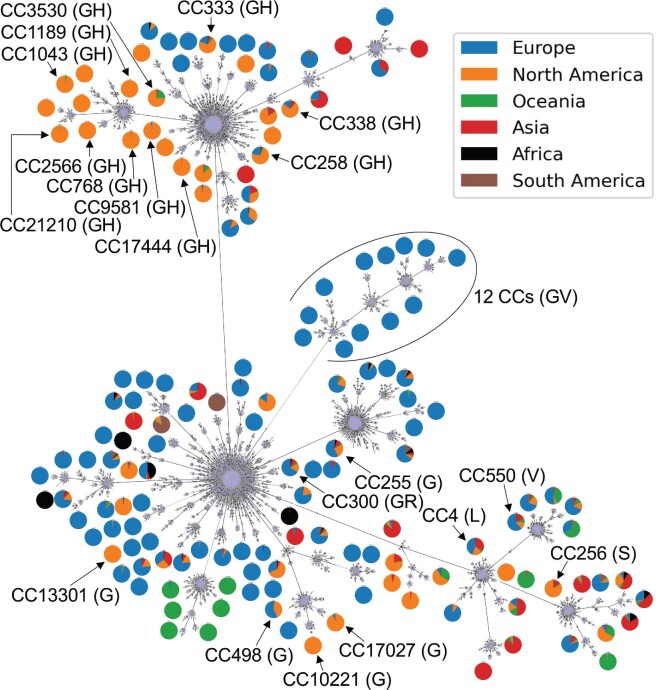
Global SARS-CoV-2 Diversity. MST from goeBURST of the 35,010 STs showing the 154 CCs identified in the data set. Only the most common 20 CCs in the 16 states are shown in black. The pie charts show the percentage of genomes from the different geographic regions in each CC. The numbers of STs and CCs in the figure are from GNUVID v2.0.

Next, GNUVID provides rapid insights into putative patterns of that can be further analyzed with other methods that account for phylogenetic uncertainty and unsampled diversity (Lemey et al. 2020; Dellicour et al. 2021; Hong et al. 2021). We used the GNUVID classifications system to assay for patterns of global spread of different CCs. When the global region of origin for each genome sequence was mapped to each CC, there was a strong association of later emerging CCs with certain geographical locations, possibly reflecting relative containment after international travel restrictions (fig. 2). To obtain an up-to-date picture of virus diversity in the United States, we analyzed 107,414 high coverage genomes (isolation dates between December 2019 to October 20, 2020) from the GISAID (supplementary table 1, Supplementary Material online). There were 26,528 genomes isolated in the United States in this data set that belong to 87 of 154 CCs. Strikingly, 35% of the genomes belong to CC258 (GISAID clade GH) and 75% of the genomes are represented by just ten CCs (CC4, 255, 256, 258, 300, 498 768, 3,530, 10,221, and 21,210). Moreover, 72% (63/87) of the CCs (representing 82% of the genomes) had the spike D614G mutation that has been associated with increased spread (Korber et al. 2020). Interestingly, none of the US genomes were associated with any of the 12 CCs (26,377, 26,754, 27,693, 27,950, 28,012, 28,825, 29,259, 29,310, 30,362, 31,179, 31,744, and 31,942) that have the spike protein A222V mutation (GISAID clade GV; Hodcroft et al. 2020). Ten of the 12 CCs with the A222V mutation were isolated only from Europe whereas the two other CCs (27,693 and 27,950) had two genomes from Hong Kong and six from New Zealand, respectively. This shows a strong association of this clade with Europe.

The relative proportions of STs or CCs isolated and sequenced may be a highly biased statistic that is contingent upon where the isolate comes from, the decision to sequence its genome, and the local capacity to sequence a whole genome. Certain states (Washington, Texas, and California) clearly sequenced more genomes than the other states. Focusing on specific states may help to partially ameliorate this bias, and we chose to focus on 16 states (Washington [WA], Texas [TX], California [CA], Wisconsin [WI], New York [NY], Michigan [MI], Minnesota [MN], Louisiana [LA], Utah [UT], Virginia [VA], Florida [FL], Oregon [OR], Massachusetts [MA], New Mexico [NM], Maryland [MD], and Connecticut [CT]) with at least 200 genomes in the studied time period, representing 92.6% (24,565/26,528) of all viral genomes available from the United States at that time. The most common 20 CCs in these states, representing 86.5% (21,261/24,565) of the genomes, are shown in figure 2.

**Fig. 3.**
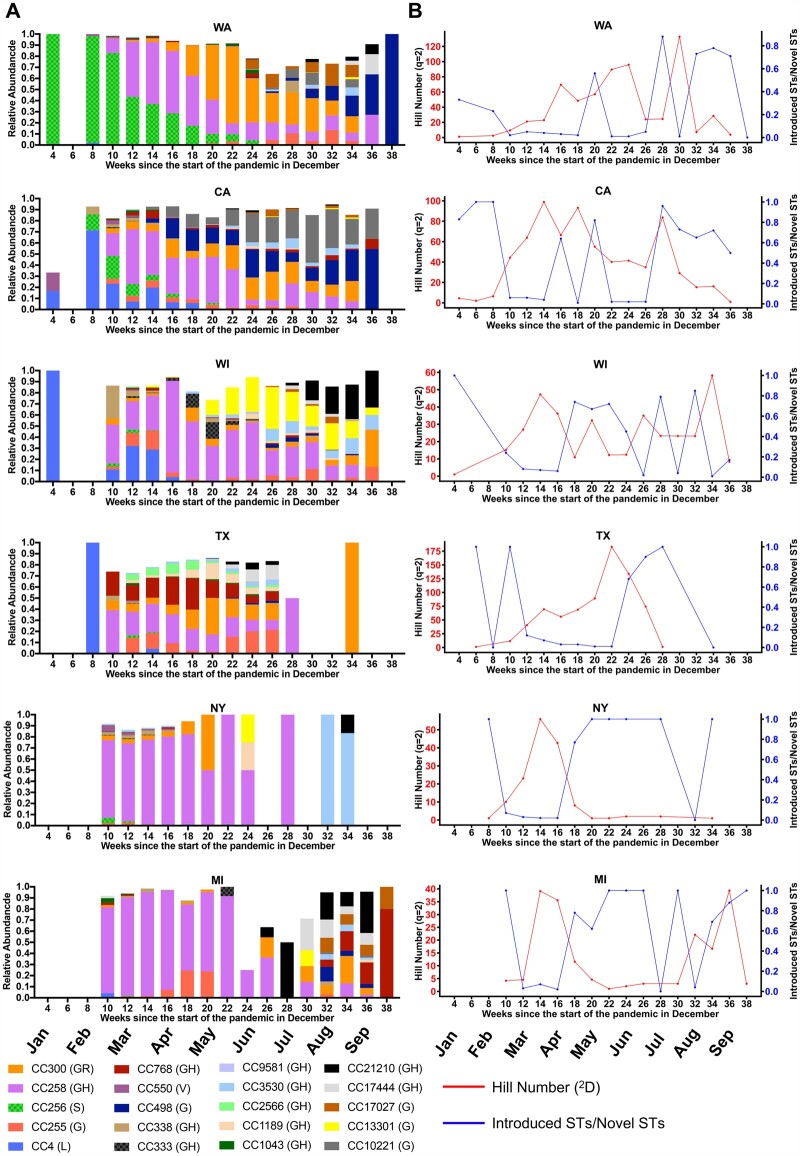
SARS-CoV-2 diversity in six states over time. (*A*) Temporal plots of circulating CCs and corresponding GISAID clade in parentheses in six different states (Washington [WA], California [CA], Wisconsin [WI], Texas [TX], New York [NY], and Michigan [MI]). The visualizations were limited to the 20 most common CCs. (*B*) Diversity of STs in the six states over time is represented for each 2-week time period in the following ratios: 1) Effective diversity (Hill number equivalent [^2^D] of Simpson index [^2^H]) (red). 2) Number of STs new to a state that were previously isolated and sequenced outside a state divided by the number of STs not seen previously in a state (blue). The plots represented data for each 2-week time period since the start of the pandemic in December 2019.

Because we included collection dates for each genomic sequence, we can use STs and CCs to better understand the emergence and replacement of certain lineages and viral diversity in geographical regions over time. Figure 3*A* and supplementary figure 3, Supplementary Material online show temporal plots of the most common 20 CCs in 16 states. In WA, the earlier introduction CC256 (GISAID clade S) was replaced by CC258 (GISAID clade GH), perhaps by introduction from the East Coast or Europe (Bedford et al. 2020; Deng et al. 2020). CC258 was then replaced by CC300 (GISAID clade GR) and subsequently by CC498 (GISAID clade G).

In the neighboring state CA, a different pattern was seen in the early pandemic where the lineage found early on in WA, CC256, only represented 20% of sequenced genomes at its most prevalent (March 1–15) whereas CC4 (GISAID clade L) was the dominant variant, which was then replaced by CC258. Interestingly, a locally emerged variant CC10221 (GISAID clade G), probably from CC498, increased in abundance over time and then was likely exported to OR and NM (supplementary figure 3, Supplementary Material online). A similar pattern was seen in WI where a local variant CC13301 increased in abundance over time and then appeared to spread to other states (NY, MI, MA, and MN). In TX, multiple diverse CCs persisted in the population until mid-July.

In NY, a different pattern was seen with CC258 being persistently dominant. However, a more granular view of STs, not CCs, in New York shows a shifting epidemiology with ST258 declining and the rise of closely related single and DLVs of ST258 reflecting local diversification (supplementary fig. 4, Supplementary Material online).

In MI, CC258 was the predominant strain until the summer when it gave way to a more diverse group of isolates. Similarly, in states like VA, CT, NM, and LA mostly one predominant CC is seen over time, whereas in other states like UT, FL, OR, MA, MD, and MN a diverse pattern of multiple CCs was noticed (supplementary fig. 3, Supplementary Material online).

The expansions and contractions in the temporal plots over time could be due to locally generated diversity (mutation) and/or introductions from other states or overseas. To better understand the source of ST diversity over time, we calculated indices reflecting effective circulating diversity as well as proportions of new STs in each state, and inferred domestic or global introductions and exportations based on previous observations in other locations or subsequent observations in other geographical locations (fig. 3*B*, table 3, and supplementary fig. 5, Supplementary Material online). To infer introductions, we required that exactly the same ST was seen at least 10 days prior in some other geographical location. For exportations, we required an ST to be seen first in the state in question at least 10 days prior to being seen anywhere else.

**Table 3:** Number of Genomes, STs, Simpson index, Hill Number, Introductions, and Exportations for 16 US States

State	Genomes (STs)	Simpson Index (^2^H)	Hill Number (^2^D)	Nonintroductions	Introductions (United States)	Exportations
WA	3,960 (1,887)	0.987	77	1,817	44 (26)	19
TX	2,167 (1,299)	0.997	319	1,258	31 (16)	17
CA	1,984 (1,236)	0.997	296	1,173	35 (19)	7
NY	1,483 (825)	0.960	25	766	25 (9)	26
MN	1,107 (522)	0.988	81	470	29 (17)	12
WI	954 (574)	0.993	147	529	26 (15)	8
VA	908 (543)	0.994	165	511	18 (13)	4
LA	850 (416)	0.988	85	397	10 (10)	1
MI	795 (416)	0.889	9	384	16 (5)	9
FL	750 (519)	0.995	215	474	29 (18)	6
OR	531 (343)	0.995	190	320	19 (14)	5
UT	350 (216)	0.992	123	204	8 (4)	2
MA	336 (170)	0.940	17	144	17 (12)	2
MD	196 (145)	0.987	76	134	8 (4)	2
NM	162 (109)	0.987	80	103	3 (1)	0
CT	154 (101)	0.964	28	84	12 (8)	0

The results of this analysis showed distinct patterns in different states with evidence supporting introductions usually outweighing evidence supporting exportations (table 3). Interestingly, NY has the highest number of putative exportations (*n* = 26), which was almost equal to the number of putative importations (*n* = 25) potentially reflecting its role as a hub driving the initial pandemic. In most states, there was a high amount of diversity that had no evidence of being introduced, which may signal problems with sampling, or perhaps that local mutation is a strong force in generating diversity. This type of analysis gives a quick and intuitive way to look at virus transmission between regions that can then be further tested with more sophisticated phylodynamic pipelines for quantifying transmission (Dellicour et al. 2020; Seemann et al. 2020).

**Fig. 4.**
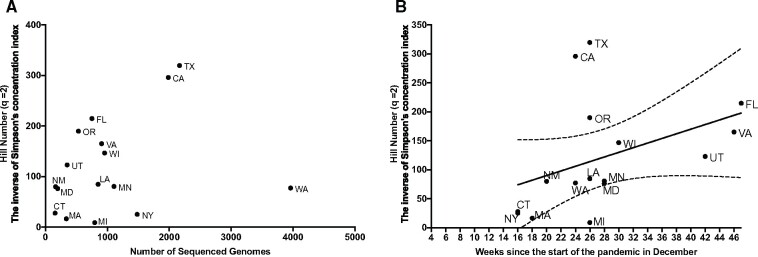
Effective Diversity of STs in 16 states. (*A*) The Hill number equivalent (^2^D) of Simpson index (^2^H) is on the *y* axis. Total number of genomes sequenced on the *x* axis. (*B*) Effective diversity (Hill number ^2^D) plotted against the week when state-wide mask mandate was imposed. Florida (FL) has no mask mandate so it was plotted at the end of the *x* axis. The solid and dashed lines show the linear regression fit and 95% confidence interval, respectively. The 16 different states are Washington (WA), California (CA), Wisconsin (WI), Texas (TX), New York (NY), Michigan (MI), Utah (UT), Virginia (VA), Florida (FL), Oregon (OR), Massachusetts (MA), New Mexico (NM), Maryland (MD), Connecticut (CT), Minnesota (MN), and Louisiana (LA). The plot represented data for each 2-week time period since the start of the pandemic in December 2019.

GNUVID classification lends itself to assessments of the ecological diversity of circulating virus. To understand the viral diversity within and between states, we calculated Hill numbers for all genomes from each state and over time in each state (fig. 4*A* and table 3). Hill numbers are a diversity metric used widely in ecological studies that express effective diversity in units of STs, and they are less prone to biases introduced by incomplete or biased sampling (Alberdi and Gilbert 2019). Recognizing that our sample was not drawn from a systematically or evenly sampled data set, we chose to use a Hill number metric (*q* = 2) that emphasizes abundant taxa in estimating the effective diversity. Several other metrics such as the Shannon Index and a normalized richness index were highly dependent on the number of sampled genomes from each state. Hill numbers based on STs varied widely by state, with TX showing the highest diversity and MI the lowest (figs 3*B* and 4 and table 3). Interestingly, there is a correlation (*R*^2^ = 0.1625) between effective diversity and when a state-wide mask mandate was imposed (fig. 4*B*).

Higher effective diversity may signal increased introduction of variants or increased local generation of new STs. To attempt to discriminate between these processes, we calculated the effective diversity over time in each state and compared this to the proportion of novel variants that were determined to be introductions (fig. 3*B* and supplementary fig. 5, Supplementary Material online). In most states, initially high numbers of introductions were followed by a drop in the relative proportion of introductions as states began to impose restrictions in March 2020. In some states, the proportion of introductions also appears to increase over the summer of 2020 as states eased regulations. Interestingly effective diversity also appeared to be associated with peaks in the number of cases (supplementary fig. 6, Supplementary Material online) in several states, especially New York, but more data will be needed to assess the connection between effective diversity and numbers of cases.

One advantage of using the GNUVID tool to type new SARS-CoV-2 genomes is that it provides more resolution for tracing virus variants of concern and interest. For instance, in the most updated version of GNUVID v2.3 (genomes included until June 21, 2021), there are 2,888 CCs and 1,346 of them represent the Alpha variant (B.1.1.7). Similarly, the Delta variant is represented by 47 distinct CCs. This adds granularity for investigating outbreaks, local patterns, and emerging variants within a particular lineage (Moustafa et al. 2021).

Although our wgMLST approach is rapid and robust, it has several limitations. Because a change in any allele creates a new ST our method may accumulate and count “unnecessary” STs that have been seen only once or may be due to a sequencing error. Sequencing errors may result in erroneous alleles, and masking known problematic sites before input may prevent this issue (Maio et al. 2020). This problem is also partially ameliorated by the use of the CC definition, which allows some variability amongst the members of a group, as well as the use of only high-quality sequences. As mentioned above, a large number of STs also may allow more granular approaches for tracking new lineages. Another limitation is the stability of the classification system, while ST designation is 100% stable, some STs may be reassigned to new CCs as clones expand epidemiologically. However, this aspect may also reflect a dynamic strength as circulating viruses emerge and replace older lineages.

Perhaps the most important limitation of our classification system is that it is limited by the quality and extent of the genomes in the GISAID database. This is also reflected as a major limitation associated with the epidemiological and diversity inferences reported here. Uneven or biased sampling could lead to both inaccurate statements of the direction or origin of import/export events, and the source and quantification of diversity. The regular update of GNUVID with more genomes and the use of diversity statistics that emphasize more predominant variants and address sampling bias such as Hill numbers may help ameliorate this problem, but it seems clear that well-designed sampling strategies are needed to confidently understand ecological dynamics for SARS-CoV-2.

## Conclusion

The genomic epidemiology of the 69,686 SARS-CoV-2 isolates studied here show that 154 CCs have circulated globally and that more than half of these have been dynamically spreading through the US population with waves of changing diversity. Our tool (GNUVID) allows for fast sequence typing and clustering of whole-genome sequences in a rapidly changing pandemic. As illustrated above, this can be used to temporally track emerging clones, identify the likely origin of viruses, and understand circulating diversity.

## Materials and Methods

All SARS-CoV-2 genomes (*n* = 110,953) that were complete and had high coverage were downloaded from GISAID (Shu and McCauley 2017) on October 20, 2020. Genomes had to be at least 29,000 bp in length and have fewer than 1% “N”s. The ten ORFs were identified in the genomes using blastn (Altschul et al. 1990) and any genome that had any ambiguity or degenerate bases (any base other than A, T, G, and C) in the ten ORFs was excluded. The remaining 69,686 genomes (supplementary table 1, Supplementary Material online) were fed to the GNUVID tool v2.0 in a time-ordered queue (first-collected to last-collected), which assigned an ST profile to each genome. The identified STs by GNUVID were fed into the PHYLOViZ tool (Nascimento et al. 2017) to identify CCs at the DLV level using the goeBURST MST (Feil et al. 2004; Francisco et al. 2009). CCs were mapped back to the STs using a custom script. Pie charts were plotted using a custom script. The sci-kit learn implementation of Random Forest was then used to train a model. The model was trained using 53,565 SARS-CoV-2 sequences from GISAID representing the 154 CCs. Briefly, the 53,565 genomes were aligned to MN908947.3 (Wu et al. 2020) to generate a multiple sequence alignment using MAFFT’s FFT-NS-2 algorithm (Katoh et al. 2002) (options: –add –keeplength). The 5ʹ and 3ʹ untranslated regions were masked in the alignment file using a custom script. Variant positions were then called using snp-sites (Page et al. 2016) (options: -o -v). The 15,136 variant positions (features) matrix of the 53,565 CC-labeled genomes were then one-hot encoded, where each SNP is replaced with a binary vector, and 70% the genomes representing the 154 CCs were used to train a random forest classifier (n_estimators = 10) in Scikit-learn. The remaining 30% were used for testing (Pedregosa et al. 2011). The prediction capability of the model was evaluated according to four statistics (accuracy, precision, recall, and F-score).

To compare the speed of GNUVID workflow against other techniques, we used a data set of 25,807 high-quality GISAID genomes that are part of the GNUVID August database release and have an assigned CC and date of isolation (supplementary table 1, Supplementary Material online). This data set was used to estimate the time for GNUVID workflow, building an ML tree using IQ-TREE or MST using grapetree or Phyloviz (table 1; Nascimento et al. 2017; Zhou et al. 2018; Minh et al. 2020). The GNUVID analysis was done on a MacBook Pro desktop computer with a single processor (3.3 GHz Intel Core i7) and 16 GB of RAM, and the run time was recorded. The ORFs cutting step was done with blastn (Altschul et al. 1990) and time was recorded. The ORFs were then compressed using GNUVID and time was recoded. The alignment for both the ML and MST was done using minimap2 (options: -a -x asm5; Li 2018). The 5ʹ and 3ʹ untranslated regions were masked in the alignment file using a custom script. Variant positions were then called using snp-sites (Page et al. 2016) (options: -o -v). The IQ-Tree step (options: -m HKY -B 1000 –nmax 100 -T 8) was run with eight processing cores and 128 Gb of memory. The grapetree MST step for the SNP matrix was run with one processing core and 47 Gb of memory (option: -m MSTree).

To compare the speed of querying using GNUVID exact matching/random forest model and Pangolin’s decision tree model, we randomly selected 1,000 genomes from the GNUVID October 2020 database (supplementary table 1, Supplementary Material online). We also randomly selected 1,000 new genomes from GISAID that were new to both GNUVID and Pangolin (supplementary table 1, Supplementary Material online). The two data sets were queried using GNUVID v2.2 and Pangolin (lineage version 01/22/2021) three times on a MacBook Pro desktop computer with a single processor (3.3 GHz Intel Core i7) and 16 GB of RAM, and the run time was recorded.

To show the relationship between our typing scheme and phylogeny, we used a Global phylogeny of SARS-CoV-2 sequences from GISAID (last accessed November 13, 2020). The tree uses an alignment of 52,747 high-quality genomes (Lanfear and Mansfield. 2020). The tree and the ten most common CCs were visualized in iTOL (Letunic and Bork 2019). We assigned a Pangolin lineage (Rambaut et al. 2020; O’Toole et al. 2021) and GISAID clade to each genome of the 53,565 genomes using the metadata details available on GISAID. We then compared the composition of each CC and calculated the percentage of the predominant clade/lineage in each CC (supplementary table 1, Supplementary Material online).

To measure the consistency of taxonomy with phylogeny, we calculated the retention index. For each of the 25,170 genomes from the August GNUVID database that were present in this tree, we coded CC and Pangolin lineage as character states using custom scripts. We then calculated the retention index using the phangorn R package (Schliep 2011).

To measure introductions and exportations from each of 16 states, we used a total of 107,414 genomes, which was the total number of genomes available in October 2020 that had a date of isolation and could be assigned to a CC (supplementary table 1, Supplementary Material online). Putative introductions were defined as an exact ST that was isolated somewhere else at least 10 days before the first date of isolation in the state in question. Exportations were defined as STs that were first isolated in the state in question and then isolated subsequently somewhere else at least 10 days later.

We used the same 107,414 genomes to compare diversity between states and in each state over time. We first calculated the Simpson index (Simpson 1949) for each state and time period. To measure effective diversity in units of STs, we then transformed Simpson index (^2^H) to a Hill number (^2^D), which is the multiplicative inverse of the Simpson index (Alberdi and Gilbert 2019). The Hill number is described as the effective number of STs (or CCs) of equally abundant STs (or CCs) that are needed to give the same diversity (Hill 1973; Jost 2006). We recorded the dates of state-wide mask mandates as the dates when face covering was required in indoor public spaces and in outdoor public spaces when social distancing is not possible (Abbott 2020; Allen 2020; Angell 2020; Baker 2020; Cuomo 2020; Edwards 2020; Evers 2020; Hogan 2020; Inslee 2020; Kunkel 2020; Lamont 2020; Northam 2020; Saunders 2020; Walz 2020; Whitmer 2020). The state-wide mandate dates used were WA (6/26/20), CA (6/18/20), TX (7/3/20), WI (8/1/20), NY (4/17/20), MI (7/10/20), LA (7/11/20), FL (no mandate), MN (7/25/20), NM (5/16/20), OR (7/13/20), MA (5/6/20), MD (7/31/20), VA (12/14/20), UT (11/9/20), and CT (4/17/20). The plots for number of confirmed cases in the 16 states were obtained from publicly available data in the Johns Hopkins University dashboard (Dong et al. 2020).

The GNUVID database will be updated regularly with newly added high-quality genomes from GISAID (Shu and McCauley 2017). Commands used are in supplementary methods, Supplementary Material online. All the scripts are available from the authors and https://github.com/ahmedmagds/GNUVID (Moustafa and Planet 2020a). The current version of GNUVID is v2.3 and can be installed through Bioconda (Grüning et al. 2018).

## Supplementary Material

Supplementary data are available at *Genome Biology and Evolution* online.

## Supplementary Material

evab197_Supplementary_Data
